# Single Tube, High Throughput Cloning of Inverted Repeat Constructs for Double-Stranded RNA Expression

**DOI:** 10.1371/journal.pone.0007205

**Published:** 2009-09-28

**Authors:** Brian Hauge, Christopher Oggero, Nicole Nguyen, Changlin Fu, Fenggao Dong

**Affiliations:** Biotechnology Monsanto Company, St. Louis, Missouri, United States of America; University of Massachusetts Amherst, United States of America

## Abstract

**Background:**

RNA interference (RNAi) has emerged as a powerful tool for the targeted knockout of genes for functional genomics, system biology studies and drug discovery applications. To meet the requirements for high throughput screening in plants we have developed a new method for the rapid assembly of inverted repeat-containing constructs for the *in vivo* production of dsRNAs.

**Methodology/Principal Findings:**

The procedure that we describe is based on tagging the sense and antisense fragments with unique single-stranded (ss) tails which are then assembled in a single tube Ligase Independent Cloning (LIC) reaction. Since the assembly reaction is based on the annealing of unique complementary single stranded tails which can only assemble in one orientation, greater than ninety percent of the resultant clones contain the desired insert.

**Conclusion/Significance:**

Our single-tube reaction provides a highly efficient method for the assembly of inverted repeat constructs for gene suppression applications. The single tube assembly is directional, highly efficient and readily adapted for high throughput applications.

## Introduction

RNAi describes a mechanism of post-transcriptional gene silencing where dsRNAs trigger the sequence-specific degradation of mRNA [1]. The dsRNAs are processed into 21–24 nucleotide (nt) short interfering (si) RNA duplexes by the RNAse III-type DICER enzymes. The small duplexes are incorporated into a siRNA-induced silencing complex (RISC) which catalyzes the sequence-specific cleavage of the target mRNA. RNAi has been described in eukaryotes from fission yeast to mammals, and is thought to have evolved as a viral defense mechanism. The power and utility of RNAi to suppress virtually any gene of known sequence has lead to its rapid adoption as an essential tool for understanding gene function and the elucidation of biological processes. RNAi offers the ability to target specific sequences within a gene, to target gene families and redundant genes, to target multiple members of a given pathway, and to produce graded levels of knock-downs and knock-outs.

DsRNAs can be produced *in vivo* by either convergent transcription [2], [3] or by transcription of a palindrome which folds back to produce a molecule with an extended double stranded region [4]. While technically a true dsRNA consists of two molecules which are not covalently linked, we will refer to the stem formed by the palindrome as dsRNA. Of the two methods, the use of vectors harboring palindromic sequences are more widely employed since they provide a highly efficient means for production of dsRNA [5] and are not complicated by issues such as transcriptional interference [6], [7]. The cloning of palindromes is frequently complicated by difficulties associated with cloning inverted repeats [8], [9] and may require sequential steps to clone the respective arms of the palindrome [10]. To accommodate high throughput cloning for the expression of dsRNAs we have developed a simple efficient LIC-based method for the cloning of inverted repeats. The method is based on PCR amplification of a target sequence with two sets of primers to generate a sense and an antisense fragment. Each of the fragments is flanked by unique sequence tags for directional assembly. To create the non-self complementary single-stranded (ss) tails for LIC assembly, the fragments are amplified with universal primers containing deoxyuridine (dU) followed by uracil DNA glycosylase (UDG) digestion [11]. For cloning, the ss-tailed sense and antisense fragments are mixed with an expression vector harboring the complementary ss-sequences and allowed to anneal. The entire cloning process from PCR amplification to E *coli* transformation can be completed in a single day. We have successfully employed this method for the cloning of hundreds of palindromic arms ranging from 25 to >1000 nt. and based on sequence confirmation >90% of the resultant clones have the expected configuration.

## Materials and Methods

### Bacterial strains and growth conditions

Annealed products were introduced into *Escherichia coli* strain DH10B [genotype: F^-^ *mcrA* Δ(*mrr*-*hsdRMS*-*mcrBC*) Φ80d*lac*ZΔM15 Δ*lacX74 deoR recA1 araD139* Δ(*ara leu*) 7697 *galU galK rpsL endA1 nupG*] (Invitrogen, La Jolla, CA) or SURE cells [genotype: endA1 glnV44 thi-1 gyrA96 relA1 lac recB recJ sbcC umuC::Tn5 uvrC e14- Δ(mcrCB-hsdSMR-mrr)171 F′(proAB^+^ lacI^q^ lacZΔM15 Tn10)] by either electroporation or by chemical transformation [12]. Cells were propagated in LB medium containing the appropriate antibiotic (from Sigma, St. Louis, MO).

### Enzymes and reagents

Restriction enzymes, nicking enzyme and UDG were purchased from New England BioLabs (Beverly, MA). KOD Hot Start DNA polymerase and Expand High Fidelity^PLUS^ PCR System were purchased from Novagen (Madison, WI) and Roche (Indianapolis, IA), respectively. DNA and PCR product purification kits were purchased from Qiagen (Germany).

### First round PCR

The first round of PCR is performed using gene-specific primers tethered to universal tails. The nucleotide sequences of the tails are presented in Table 1. PCR reactions were performed in a 50 µL reaction volume containing 5 µL of 10X KOD buffer, 5 µL dNTPs (2 mM each), 2 µL of each primer (5 µM), 10 µL of 5 M Betaine, 2 µL MgSO_4_, 0.5 U KOD Hot Start DNA polymerase and 2.0 µL DNA template (15 ng/µL). Thermocycling was preformed on a MJ Research Tetrad Thermal Cycler PTC-225 using the following conditions: 94°C for 2 min, 10 cycles at 94°C for 15 sec, 65°C for 30 sec (minus 1°C/cycle) and 72°C for 1 min, followed by 25 cycles of 94°C for 15 sec, 55°C for 30 sec and 72°C for 1 min, followed by 1 cycle at 72°C for 2 min.

**Table 1:** Primers for LIC-based assembly.

PCR reaction	Primer	Sequence 5′-3′
1^st^ round anti-sense	a5′	GCAGTCGCTGTCGTTACC
	a3′	AGCGTTAACGCGATCGCAGTACTT
1^st^ round sense	s5′	CTCCTCATCCACGCGGCCGCCTGCAGGAGC
	s3′	GCGAGTACCGCTGGGTTCTA
1^st^ round anti-sense	dUa5′	GCAGUCGCUGTCGTUACC
	dUa3′	AGCGTUAACGCGAUCGCAGUACTT
1^st^ round sense	dUs5′	ACTGCGAUCGCGUTAACGCUTTATC
	dUs3′	GCGAGUACCGCUGGGTUCTA

PCR primers used for the assembly of palindromic RNAi constructs The first round PCR sequences correspond to the 5′ tags with are tethered to the gene specific primer sequences The second round PCR primers contain dU residues used to generate ss tails The vector overhang depicts the single stranded regions of the vector which are generated following HpaI and NbBbvCI digestion The left and right ss vector overhangs are complementary to the 3′ tails generated by UDG cleavage of dUa5′ and dUs3′ of their respective PCR products.

### Second round of PCR with uracil containing primers

**Figure 1:**
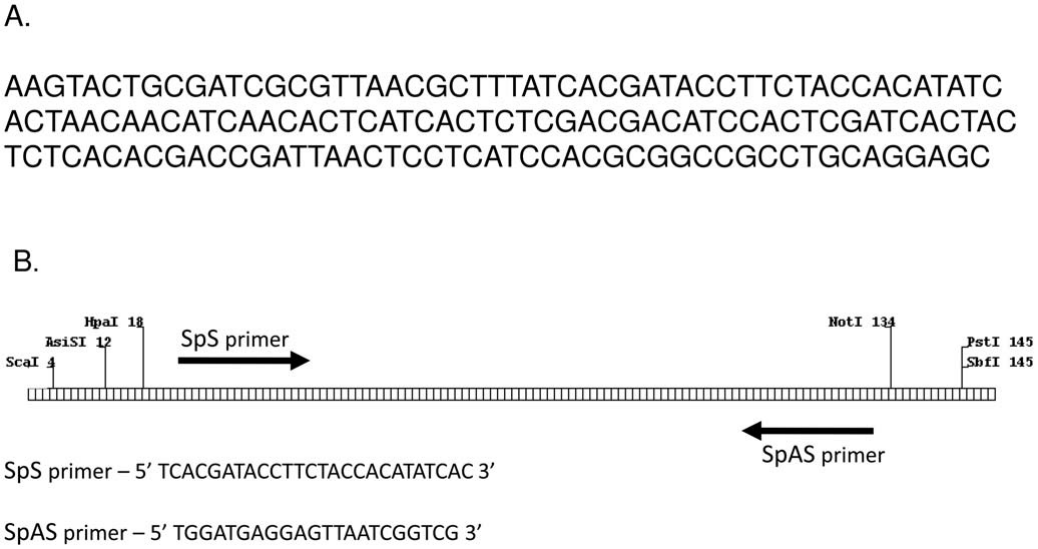
Universal loop facilitates DNA sequence confirmation of the insert. DNA sequence of the universal loop is shown in panel A Panel B depicts the restriction sites used to linearize the construct for sequence confirmation. The relative position of the sequencing primers is depicted and their sequences are provided.

A second round of amplification is performed to introduce uracil residues into the universal tails. During this round of PCR the spacer sequence (Figure 1) is tethered to the sense product using SOE (Splicing by Overlap Extension) PCR [13]. The sense and antisense amplifications are carried out in separate 50 µl reactions containing 10 µL of 5X buffer with MgCl_2_, 5 µL dNTPs (2 mM each), 2 µL of each primer (5 µM), 0.5 U Expand High Fidelity^PLUS^ enzyme, and 1.0 µL 1^st^ round PCR product. The spacer has been cloned into a pUC19 for either PCR amplification or digestion followed by gel purification. For SOE-PCR 20 ng of spacer is added to the sense PCR reaction. The amplification conditions for the antisense reaction are as follows: 94°C for 2 min, 16 cycles at 94°C for 15 sec, 50°C for 30 sec and 72°C for 1 min, followed by an additional extension at 72°C for 2 min. For sense reactions the amplifications were carried out using the following conditions: 94°C for 2 min, 24 cycles at 94°C for 15 sec, 50°C for 30 sec and 72°C for 1 min, followed by an additional extension at 72°C for 2 min.

### LIC cloning reaction

The cloning vector is prepared by digestion with HpaI and the nicking enzyme Nb.BbvC I. The ends of the PCR products are made single stranded by treatment with UDG as follows: add 1.5 µL 5X PCR buffer with MgCl_2_ (from the Expand High Fidelity^PLUS^ PCR system), 1 µL of UDG (1 U), 5 µL each of the sense and antisense products, bring to 15 µl with dH_2_O and incubate at 37°C for 1 hour. Following the digestion add 10 ng of the linearized and nicked vector, incubate the mixture at 65°C for 5 min, cool the mixture to 37°C (1°C per second) and incubate for 1 hour at 37°C. 1 µL of the annealing reaction is used to transform E *coli*. Plasmid DNA is prepared from resulting colonies and analyzed by sequencing.

#### Screening and sequencing

Four colonies from each transformation are picked into 96-well plates containing LB medium plus the appropriate antibiotics. DNA was prepared with a Qiagen mini prep kit. 5 µl of DNA (∼100 ng/µl) is linearized by restriction enzyme digestion (the cutting site was located in the spacer region) to separate the arms of the hairpin prior to sequencing. Sequencing reactions using universal stock primers located upstream and downstream of the insert are performed according to standard operating procedures for the ABI3730. The spacer sequence and accompanying primers have been submitted to GenBank and has been assigned the following accession number; GQ245680.

## Results

**Figure 2:**
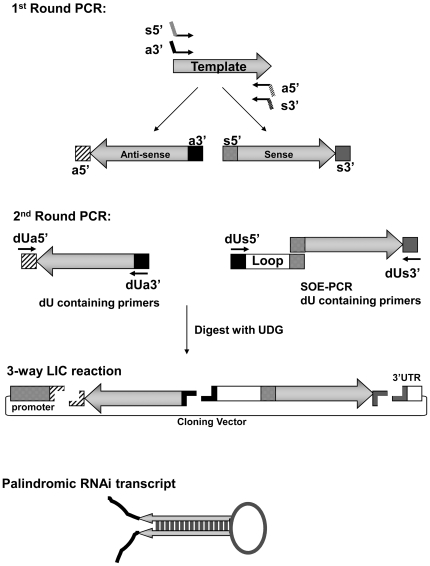
Strategy for LIC-based assembly of palindromic RNAi constructs. Four PCR primers are synthesized containing the s5′ and a3′ tags tethered to the forward primer, and the a5′ and s3′ tags tethered to the reverse primer For each target two amplifications are carried out using the a5′/a3′ primer pair and the s5′/s3′ pair to generate the antisense and sense products respectively In the second round of PCR an aliquot of each reaction is amplified using the cognate dU containing primers During the second round of PCR a universal loop is added to the sense product by SOE-PCR The loop is prepared by PCR amplification and a stock is stored at −20°C For the LIC based assembly 5 µl aliquots are mixed with 10 ng of vector, treated with UDG, heated to 65°C and allowed to anneal.

**Figure 3:**
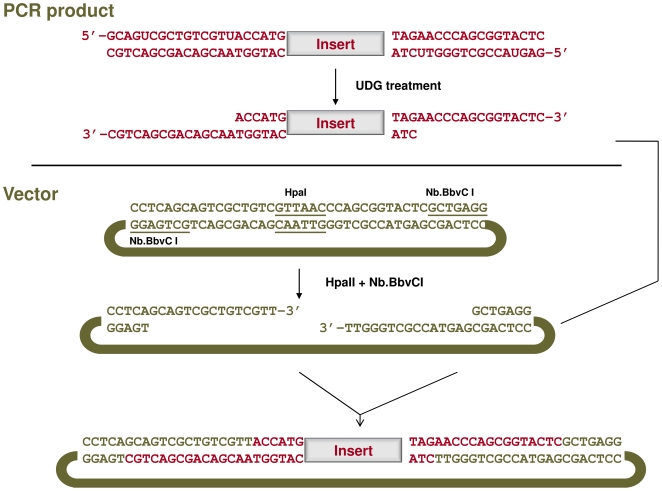
Vector production and insert annealing. Diagram depicting UDG mediated ss production of the insert and production of the complementary vector ss tails by restriction digestion (Hpa1) and nicking with the nicking endonuclease (NbBbvCI) Assembly is mediated by efficient annealing of the complementary ss tails.

The strategy for LIC-based cloning of inverted repeat constructs is outlined in Figure 2. In the first round of PCR the target sequence is amplified with two pairs of primers to generate sense and antisense products. Each of the fragments is flanked by unique sequence tags which anneal to the primers used in the second round of PCR. The second round of PCR is carried out to incorporate primers containing one or more deoxyuridine residues and to tether the spacer to the sense product using the technique of SOE- PCR [13]. The specificity for efficient directional assembly of the palindrome is provided by the tags which generate complementary ss-tails following treatment of the uracil containing products with UDG. When the products are mixed together with the vector, the complementary sequences anneal to produce the desired clone. Unlike ligations which rely on symmetrical restriction sites there is no ambiguity in the process since the products can only assemble in the pre-defined orientation. As depicted in Figure 2, the antisense product is amplified with the a5′ and a3′ tagged primers and the sense product with the s5′ and s3′ tailed primer pair. After the second round of PCR the products are mixed with the vector, treated with UDG and allowed to anneal. Vectors containing single stranded tails which are complementary to the insert are prepared by linearization with a restriction enzyme and nicking with a nicking endonuclease as shown in Figure 3. Following annealing an aliquot is used to transform E *coli*. Alternatively the reaction may be stored for weeks at −20°C prior to transformation.

In Table 2, we present the results for three projects where 174 target sequences were nominated for cloning into three different expression vectors. Of the 174 nominations, 168 (98%) produced the predicted sized products for their respective sense and antisense products following the first round of PCR. As indicated in Table 2, 160 of the paired PCR products produced the expected clone as confirmed by DNA sequencing, representing a 92% cloning success rate. If we eliminate the first round PCR failures our first pass success rate for obtaining the desired clone is 95% (160/168). It should be noted that confirmation is based on picking eight individual colonies which are prepped and submitted for DNA sequencing. Eight colonies is the number that has been established for our production pipeline to ensure that we get full length high quality sequence reads for clone confirmation. It is more efficient to pick more clones up front than to remediate if a production or technical issue arises. It should further be noted that we track our process based on the nomination and not on the individual colony, so when the first clone is confirmed the remaining sequences are not analyzed. In other words we do not track metrics as to whether eight of eight clones were correct, or if a non confirming clone is do to a cloning failure, a sequencing failure or a related issue.

**Table 2:** LIC cloning of inverted repeats into three different vectors.

Vector	Number of distinct targets	Number of 1st round PCR Products	First Round PCR Success Rate	Number of 2nd round PCR Products	Second Round PCR Success Rate	Number of Successful LIC Constructs	Cloning Success Rate
1	114	109	96%	101	93%	101	93%
2	24	24	100%	24	100%	24	100%
3	36	35	97%	35	100%	35	100%
Total	174	168	98%	163	97%	160	95%

Successful cloning is based on the availability of the desired target sequences, so failure to amplify the target sequence is a PCR failure and is not considered a cloning failure. The cloning success rate refers to the number of the desired LIC constructs attained following one round of cloning; this includes incorporation of the uracil containing primers, addition of the loop, and generation of the ss tails and annealing to the vector in the desired orientation.

**Figure 4:**
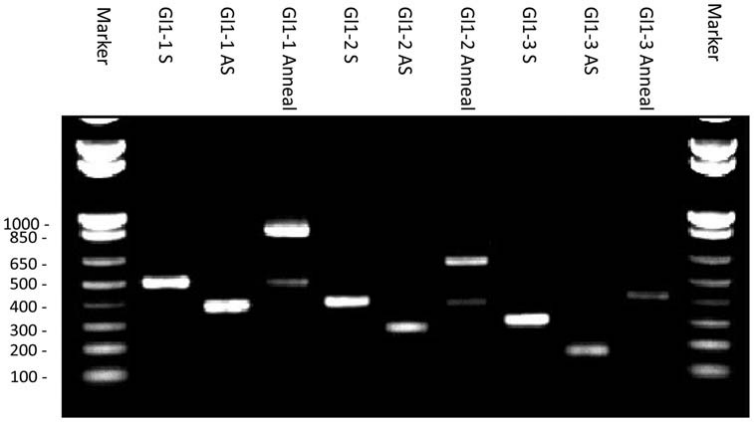
Annealing as visualized by agarose gel electrophoresis. Gel image showing the annealing of three different Gl1 RNAi constructs The sense, antisense and annealed products are labeled S, AS and Annealed respectively The sense product is larger than the antisense product since it is tethered to the 150 bp universal loop The sizes of the relevant size markers are indicated.

For smaller discovery projects cloning is sometimes performed outside of our production pipeline. An example is presented in Figure 4, where primers were designed to amplify three overlapping regions of the *Arabidopsis Gl*1 gene of 129, 231 and 345 bps. The sense products are larger than their cognate antisense products since they are tethered to a 150 bp spacer which serves as a loop separating the arms of the inverted repeat. As described above the respective forward and reverse products were mixed with vector, treated with UDG and allowed to anneal. The annealed products were run on a 1% agarose gel, and their respective sense and antisense products are run in adjacent wells. As shown in Figure 4, the sense and antisense products are efficiently annealed to produce a single product of the size predicted for their respective antisense-loop-sense products. The minor band which co-migrates with the sense-loop product represents residual non-annealed product. The annealing of the inverted repeat to the vector is not readily visible on the gel since we use a large molar excess of PCR products relative to the amount of vector used (10 ng/reaction). In our experience the LIC cloning procedure is largely insensitive to the amount of PCR product so it has not been necessary to optimize the amount of product used in the annealing reaction. If the products can be visualized on a gel they can be readily cloned.

The LIC cloning reactions were performed using two vectors each containing different expression elements (Figure 4 depicts annealing with one of the two vectors). Following transformation into E *coli* four colonies from each were picked for DNA sequence confirmation of the insert. For all six of the constructs, four-of-four of the clones contained the expected fragments assembled in the predefined orientation, and had the expected sequence (data not shown). While this example represents a small number of clones, it mirrors our results based on the cloning attempts that we have done outside of the production pipeline. The authors of this paper have produced 25 different non-pipeline inverted repeat constructs and for all 25 every colony that was picked contained the insert of interest in agreement with the >90% first pass cloning rate. While deviations from the expected sequence are observed they are restricted to point mutations and small indels which can be attributed to the fidelity of the polymerases used for the PCR amplification.

DNA sequencing of inverted repeats can be very challenging since snap back to form self dimers can interfere with polymerization. To circumvent this problem we cleave the DNA in the spacer to eliminate the self dimerization which occurs with intact molecules following thermal denaturation. The spacer was designed to contain recognition sequences for infrequent cutting restriction enzymes near the extremities of the loop, as well as sites for efficient annealing of sequencing primers. For sequence confirmation, two independent digests are performed using enzymes which cut at opposite ends of the spacer. As depicted in Figure 1, clones digested near the right end of the spacer are sequenced with the SpAS primer, while digestion near the left end facilitates sequencing with the SpS primer. The combination of the spacer specific primers and the vector specific primers allows every base in the palindrome to be sequenced from both strands for unambiguous confirmation of the entire insert. While the requirement to pre-digest the clones is more labor intensive it nonetheless proves to be very effective when sequence confirmation of the entire palindrome is required.

## Discussion

We have developed a highly efficient LIC-based method for the cloning of inverted repeats for the *in vivo* expression of dsRNAs. The method is based on PCR amplification of a target sequence with specific primers which are tethered with two pairs of universal tags which specify either the sense or antisense orientation. These products are then used directly in a second PCR reaction which is used to add uracil-containing primers to the ends of the amplicons. The second round of PCR also includes SOE-PCR which serves to add a universal spacer to the sense product. Following PCR amplification the products are treated with UDG to create the complementary single strand (ss) tails used for LIC assembly (Table 1). Cloning is achieved by mixing the ss-tailed sense and antisense products with a cloning vector that contains complementary ss-tails under conditions which promote efficient annealing of the fragments. Since each of the ss-tails is unique and complementary by design, the fragments can only assemble in the predefined orientation. Based on several hundred different clones that we have characterized virtually all inserts are in the predicted configurations. Moreover, the requirement for base pairing of long non-self complementary tails means that cloning is relatively insensitive to insert concentration (data not shown).

Vector preparation is the major factor impacting cloning efficiency. Our cloning vectors are prepared by linearization with a restriction endonuclease and by digestion with a nicking endonuclease to create the desired ss-tails. Incomplete digestion with the restriction enzyme leads to high background while incomplete digestion with the nicking endonuclease reduces the cloning efficiency. It is important that the vectors are digested to completion and if necessary gel purified to eliminate undigested products.

There are several published methods for cloning of palindromes. The pHellsgate strategy [5] is based on the widely used Gateway^TM^ technology which utilizes the bacteriophage λ site specific recombination system [14]. The pHELLSGATE vector was designed such that a single PCR product flanked by att sites is recombined simultaneously to form the two arms of the palindrome. This system is amenable to HTP cloning and the authors reported cloning efficiencies of ∼90% [4]. Limitations of the system include the requirement to flank the sequence of interest with the 25 nt recombination sites, and since there are two alternative products of the recombination reaction it is necessary to screen the clones for the desired orientation [5]. A second strategy called inverted repeat PCR (IR-PCR) is similar to our procedure in that it relies on differentially tagging antisense and sense copies of the target in two separate PCR reactions [15]. The products are then assembled by SOE-PCR in a three-way reaction and the resultant palindromic product is cloned by restriction cloning. This system does not require any specialized vectors so the palindrome can be cloned into any expression vector with a poly-linker or appropriate restriction sites [15]. Since directional cloning requires cleavage with a pair of restriction enzymes it is not possible to define a universal pair of enzymes that work for all genes, so the choice of enzymes and the primers harboring the recognition sequences may need to be defined on a gene by gene basis. We and others have observed that the presence of palindromes in the cloning vector can negatively affect replication of the plasmid in E *coli* [9], [10], [and 11] which can augment any inefficiency in the cloning process. Therefore cloning of palindromes frequently requires screening a large number of candidate clones.

In our cloning strategy depicted in Figure 2, we have chosen to incorporate a universal spacer to separate the arms of the palindrome. The use of a 150 nt universal spacer allows us to standardize our processes for downstream characterization. The spacer is designed to separate the arms of the palindrome for efficient cloning, to provide restriction sites for linearization of the construct and to provide unique primer binding sites for sequence confirmation of the insert. As an alternative we have assembled constructs where the spacer is simply an extension of the target sequence. In this case the PCR primers are designed so that one of the arms is longer than the other and this extension serves as the spacer. Since each construct has a unique loop universal sequencing primers can not be employed. The use of a gene specific spacer may compromise the ability to separate the two arms by simple restriction cleavage, which is a step that greatly facilitates sequence confirmation of the inserts. However, restriction sites can easily be incorporated into LIC tails to facilitate downstream sequence confirmation of the insert.

The vector requirements for our cloning system are relatively simple and allow for flexibility in the vector design. The requirements are a unique restriction enzyme recognition site which is flanked by sites for a nicking endonuclease (Figure 3). Ideally the restriction site should end with a T. The distance from the cleavage site to the nicking site should be a minimum of 12 nt which has been shown to provide efficient annealing [16]. Aside from the requirement for the recognition sequences for the restriction and nicking endonucleases, the intervening sequence can be tailored to meet the needs of the experiment.

Our LIC protocol is based on two rounds of PCR. The first round of PCR uses gene specific primers while the second round is used to incorporate the dU containing primers used to create the complementary ss-tails. We can easily incorporate dU-containing primers in the first round of PCR but this increases the cost since each individual primer pair needs to be synthesized with modified bases which are significantly more expensive than conventional primers. Since the second round utilizes universal dU-containing primers it represents a significant cost saving for high throughput applications. In addition, the second round of PCR is used to tether the loop to the sense product. However alternative strategies, such as where the loop is a simple extension of either the sense or antisense arm, are fully compatible with a single round of amplification. The decision as to whether to use a single round of PCR or two rounds is dictated by the nature of the construct and the needs of the project.

We have established a procedure which uses dU containing oligonucleotides and UDG for the generation of ss-tails, however this is only one of several methods available. An early publication describing LIC cloning exploited the 3′–5′ exonuclease activity of T4 DNA polymerase to generate 5′ ss-tails [16]. Subsequent methods have been described which use exonuclease III to produce 5′ overhangs [17] RNA/DNA primers followed by either RNAse or alkali treatment [18], [19] or the use of modified nucleotides which block the progression of DNA polymerase [20], [21], [22], [and 23]. Any one of the available methods for the generation of ss tails can be adapted for our procedure.

Cloning by annealing of unique complementary ss-tails is very efficient since the fragments can only assemble in one orientation. In our process we screen eight colonies for each LIC reaction by DNA sequencing of both arms of the palindrome and the intervening spacer. Excluding sequencing failures our success rate, defined as identifying a base perfect clone, is >90%. We are not providing absolute numbers since our process is such that when the first base perfect clone is identified it is advanced and characterization of the remaining clones is terminated. Our experience to date is that if we can successfully amplify the sense and antisense fragments we have always been able to generate the clone of interest.

RNAi has emerged as an essential laboratory tool for elucidation of gene function and has rapidly been adopted for functional genomics, pathway analysis and target validation. Accordingly there is an ever increasing need for high throughput methodologies for the construction of vectors for gene suppression applications. The LIC cloning method which we have developed provides a means to rapidly clone palindrome containing constructs for the production of dsRNAs. The method is simple, inexpensive, and highly amenable to automation.
